# Mechanism and application of fermentation to remove beany flavor from plant-based meat analogs: A mini review

**DOI:** 10.3389/fmicb.2022.1070773

**Published:** 2022-12-01

**Authors:** Anqi Tao, Hongyu Zhang, Junnan Duan, Ying Xiao, Yao Liu, Jianwei Li, Jieyu Huang, Tian Zhong, Xi Yu

**Affiliations:** ^1^Faculty of Medicine, Macau University of Science and Technology, Macau, Macau SAR, China; ^2^Guangdong-Hong Kong-Macao Joint Laboratory for Contaminants Exposure and Health, School of Environmental Science and Engineering, Institute of Environmental Health and Pollution Control, Guangdong University of Technology, Guangzhou, China; ^3^School of Pharmacy and Food Science, Zhuhai College of Science and Technology, Zhuhai, China; ^4^Macau Uni-Win Biotechnology Co., Ltd, Macau, Macau SAR, China

**Keywords:** beany-flavor, plant-based meat analogs, fermentation, soy proteins, pea proteins, off-flavor

## Abstract

Over the past few decades, there has been a noticeable surge in the market of plant-based meat analogs (PBMA). Such popularity stems from their environmentally friendly production procedures as well as their positive health effects. In order to meet the market demand, it is necessary to look for plant protein processing techniques that can help them match the quality of conventional meat protein from the aspects of sensory, quality and functionality. Bean proteins are ideal options for PBMA with their easy accessibility, high nutrient-density and reasonable price. However, the high polyunsaturated lipids content of beans inevitably leads to the unpleasant beany flavor of soy protein products, which severely affects the promotion of soy protein-based PBMA. In order to solve this issue, various methods including bleaching, enzyme and fermentation etc. are developed. Among these, fermentation is widely investigated due to its high efficiency, less harm to the protein matrix, targeted performance and low budget. In addition, proper utilization of microbiome during the fermentation process not only reduces the unpleasant beany flavors, but also enhances the aroma profile of the final product. In this review, we provide a thorough and succinct overview of the mechanism underlying the formation and elimination of beany flavor with associated fermentation process. The pros and cons of typical fermentation technologies for removing beany flavors are discussed in alongside with their application scenarios. Additionally, the variations among different methods are compared in terms of the strains, fermentation condition, target functionality, matrix for application, sensory perception etc.

## Introduction

The Food and Agriculture Organization of the United Nations estimates that in 2019, humans consumed about 3.25 million tons of meat; demand for meat is expected to increase by another 12% by 2029; and by 2050, demand for meat will increase by about 70%. If traditional meat production and management patterns remain unchanged, an additional 30%–50% of the land will be needed for livestock and meat production by then (Chen et al., 2019). Moreover, such situation of undersupply can be further intensified by natural disasters showing up every now and then. Animal diseases such as African swine fever and avian influenza in 2018 caused an increasing number of small and medium-sized livestock companies to exit the market. During the Coronavirus Disease 2019 (COVID-19) pandemic, the meat, poultry, and animal product processing plants were the sectors most affected, which leads to imbalance between global supply and demand for meat products (Chen and Yang, 2021). This situation has now triggered an increase in the price of traditional meat products. The global agriculture and meat industry is facing serious challenges considering factors such as increasing pressure on the natural environment, population growth, consumer trends in health and environmental protection, and food innovation (Kumar et al., 2017). To relieve the supply pressure of meat products, the development of plant-based meat analogs (PBMA) can be an effective way to address the imbalance between meat production and consumption (Taufik et al., 2019; Sun et al., 2022).

Recent PBMA research and development has been focusing on utilizing raw materials such as soy and pea proteins to mimic the flavor, smell, appearance and texture of traditional meats. It is beneficial for the whole mankind not only in terms of promoting a sustainable development, but also from a nutrition aspect (Kyriakopoulou et al., 2021). Compared to traditional meat, the energy, land, and water consumption of plant-based meat is much lowered. That is why this new star can effectively mitigate carbon emissions and moderate global environmental change (Pimentel and Pimentel, 2003). Many epidemiological studies have shown that long-term meat consumption, especially red and processed meats, increases the incidence of digestive cancer, cardiovascular disease and hypercholesterolemia (Cordelle et al., 2022). Conversely pieces of evidence support the health benefits of consuming a plant-based diet and increasing the intake of legumes. Significantly reduces the incidence of heart disease, high blood pressure, stroke, and type 2 diabetes (Polak et al., 2015).

Nevertheless, despite the many benefits of PBMA, their market share is still low at around 1% (Choudhury et al., 2020). The major bottleneck for developing ideal products which can cater the public lies in the texture as well as the taste. Other problems include high energy consumption of the production line, rough finished products and premature control technology. Currently, the key problem with the flavor of PBMA is that soy and pea proteins have an unpleasant beany-flavor which hinders consumer preference and acceptability (Mittermeier-Kleßinger et al., 2021). Flavorings are added during the production process to cover the off flavors as a current mainstream solution. However, the addition of extra seasonings may also have an impact on the overall taste and bring about adverse health issues for the consumers. Other methods to reduce the beany flavor include temperature control, enzyme treatment, acid–base treatment, supercritical CO_2_ extraction, new cultivars breeding, genetic engineering, etc. (Zhang et al., 2012). However, some of these methods have hidden risks for food safety and quality, while the high cost and extra energy consumption also bring about new problems (Kumari et al., 2015; Nedele et al., 2021; Korma et al., 2022). Therefore, searching for novel technology to remove beany off-flavors instead of covering them is significant to improve the overall taste and future development of PBMA (Sun et al., 2022).

Recently, through the effort of modern microbiologists, it is more and more revealed that fermentation using microorganisms can effectively remove the beany flavor from next-generation plant-based food products such as PBMA. Moreover, a new aromatic taste can be developed during the fermentation to mask or cover the beany flavor sometimes (Wang Z. et al., 2022). Other than that, fermentation has many additional benefits, such as adjusting the gut microbiota and remediating the detriment on the gut epithelium caused by food additives (Yu and Zuo, 2021). This review discusses the mechanism of the formation of beany odors, the mechanism of using fermentation to remove them, and the applications and the differences of various traditional and newly emerged techniques. In the end, summary and discussion are made about the possibilities to utilize and improve current fermentation technique to better develop our food for future signatured by PBMA.

## The formation of beany flavor

Unsaturated fatty acids are the main cause of beany flavor formation in legume-based foods. The formation of off-flavor compounds usually results from the oxidation of unsaturated fatty acids and the hydrolysis of lipids (Jelen, 2011). Legume seeds contain a large amount of unsaturated fatty acids, the most abundant being oleic acid, linolenic acid, and linoleic acid (Khrisanapant et al., 2019). The oxidation mechanism generally consists of three categories: automatic oxidation, photo-oxidation, and enzymatic oxidation (Wang Y. Q. et al., 2022).

### Automatic oxidation

**Figure 1:**
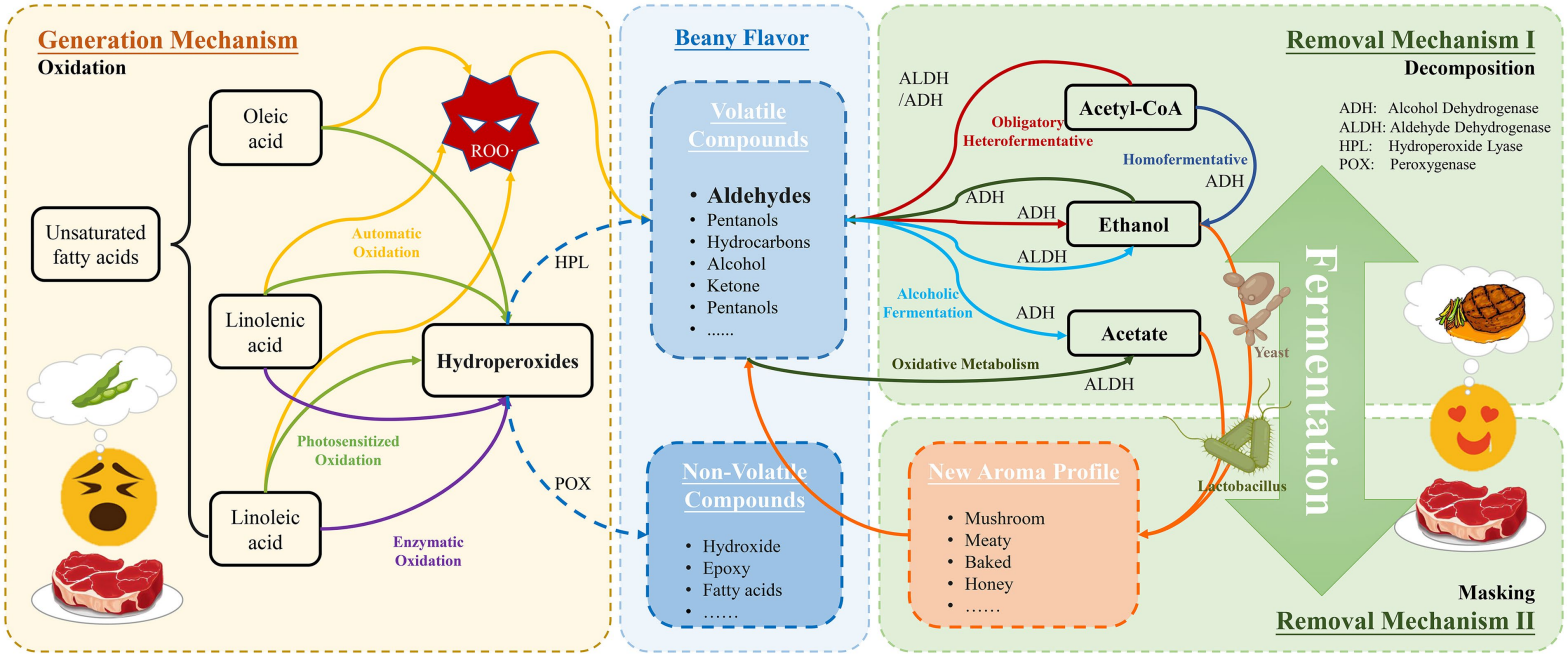
The generation and removal mechanism of beany-flavor.

As shown in the yellow route in Figure 1, automatic oxidation is a free radical chain reaction involving oxygen and unsaturated lipids (Wang Y. Q. et al., 2022). Principally, reactive singlet oxygen attacks H, forming α-methylene near the double bonds, thus forming alkyl radicals (R·). After that, further oxygen absorption leads to the formation of peroxyl radicals (ROO·) and hydrogen peroxide, which ends up with a wide range of volatile and non-volatile secondary products, odor compounds (Wang et al., 2021). Once the chain reaction starts, it is very difficult to be stopped. Therefore, preventing chain reaction initiation is the most effective way to control autoxidation.

### Photosensitized oxidation

The left green route in Figure 1 displays the photosensitized oxidation, which differs from automatic oxidation in that during photosensitized oxidation, highly reactive singlet oxygen directly attacks the double bonds of unsaturated fatty acids, resulting in oxygen binding directly to the fatty acids in the formation of hydrogen peroxide. Singlet oxygen is more significant for photosensitized oxidation in the oxidation process than automatic oxidation (Hammond and White, 2011).

### Enzymatic oxidation

As illustrated in the purple route in Figure 1, enzymatic oxidation is the main reason to produce most volatiles in legumes, which is the primary way in which off-flavor is generated. Lipoxygenase (LOX) is a major element of enzymatic oxidation (Roland et al., 2017). LOX belongs to the family of nonheme iron-containing enzymes that can effectively catalyze the deoxygenation of polyunsaturated fatty acids (PUFAs) such as linoleic acid (18:2) and linolenic acid (18:3), to produce hydroperoxyl derivative (Alhendi et al., 2018). Because oxidation reactions usually occur in specific regions and oxygen is generally bound within C9 and C13 (Gardner, 1991), Soy LOX isoenzymes are classified into three types: 9-, 13- and 9-/13-LOXs. For example, soy LOX-1 is a 13-LOX, LOX-2 is a 9-/13-LOX, and soy LOX-3 is a 9-LOX or 9-/13-LOX (Wang Y. Q. et al., 2022).

After the formation of hydrogen peroxide derivatives through three processes: automatic oxidation, photosensitized oxidation and enzymatic oxidation. As shown in Figure 1, the main volatile flavor compounds, and nonvolatile lipid oxidation products were produced through three different oxidation mechanisms (Roland et al., 2017; Zhang et al., 2020). Hydroperoxide lyase (HPL), alcohol dehydrogenase (ADH), and peroxygenase (POX) will further catalyze the generation of products and aggregation, resulting in beany flavor. Hydroperoxide lyase (HPL) reacts with hydroperoxyl derivative to form volatiles such as C6 aldehydes and C9 aldehydes, and in the presence of ADH, these aldehydes are reacted to the corresponding alcohols, such as alcohols and acids. And hydroperoxyl derivative forms non-volatile lipid oxidation products such as hydroxy, epoxy, and fatty acids in the presence of POX (Wang Y. Q. et al., 2022).

Therefore, in order to control the production of beany flavor, we have to control the oxidation and degradation of unsaturated fatty acids. In the automatic oxidation process, the concentration of oxygen is extremely important, the higher the concentration of oxygen, the faster the rate of automatic oxidation (Wang et al., 2021). On the contrary, in the photosensitized oxidation process, the oxygen concentration has little effect, and the number and distribution of unsaturated double bonds have a significant effect (Wang et al., 2020). And in the enzymatic reaction process, lox is its limiting factor (Roland et al., 2017; Wang et al., 2021).

## The mechanism underlying the fermentation removal of beany off-flavor

There are currently two major types of methods to remove beany flavor from plants. The first way is to use enzymes to decompose the components that produce the beany flavor in plants and use enzymes to decompose the precursor substances of the plants that produce the beany flavor (Zhu and Damodaran, 2018; Fischer et al., 2022). The second way is to generate new aromatic profile which can mask the original beany flavor.

### Direct decomposition of compounds with beany off-flavor

It was found that ADH and aldehyde dehydrogenase (ALDH) had the most obvious effect on the removal of beany flavor. Because the selection of compounds involved in the beany flavor included nine aldehydes, one furan, four alcohols, four ketones, three sulfides, and five pyrazines, the compound with the greatest effect on “beany” are aldehydes (Xu et al., 2020). ADH converts aldehydes to primary alcohols and ketones to secondary alcohols. ALDH converts aldehydes to carboxylic acids. Reduces beany flavor by converting odorous compounds (aldehydes and ketones) to less odorous products (alcohols and carboxylic acids; Shi et al., 2021).

As illustrated in the upper right of Figure 1, there are five main ways they remove the beany flavor: (a) The blue route illustrates the homofermentation: For the catabolism that occurs in this type of fermentation, ADH does not participate in basal metabolism, but plays a role in the mixed acid pathway. Such as *Lactobacillus acidophilus* and *Streptococcus thermophiles* (Shi et al., 2021). (b) Obligatory heterofermentative Lactic acid bacteria illustrated with the red route (LAB): such as *L. fermentum*. ADH is present in metabolism as a constitutive enzyme as well as an aldehyde/alcohol dehydrogenase (Ortakci et al., 2015; Verce et al., 2020). (c) Facultative heterofermentative LAB: Only the heterofermentative pathway is inducible. Such as *L. plantarum*. Its basic metabolism is the homofermentative pathway (Prückler et al., 2015). (d) Alcoholic fermentation illustrated with the cyan route: ADH and aldehyde dehydrogenase are present in the catabolism of the strain as constitutive enzymes. For example *Saccharomyces cerevisiae* (Mizuno et al., 2006). (e) Oxidative metabolism. “Oxidative fermentation” is the incomplete oxidation of substrates, carried by the dehydrogenases of the respiratory chain, resulting in the extracellular accumulation of oxidized products. The “oxidative system” is located in the cytoplasmic membrane and is connected to the respiratory chain, for example, *Gluconobacter suboxydans* (Deppenmeier and Ehrenreich, 2009; Lynch et al., 2019).

### Breaking down beany flavor precursors in plants

Previous studies have indicated that the beany flavor in legumes is primarily the result of interactions between lipid oxidation products, proteins, and phytochemicals. Lipid-derived off-flavors are believed to be the main cause of beany flavor. Among them, the precursors of the flavor of legumes are mainly phospholipids (PLs) and free fatty acids (FFAs).

Phospholipase can be used to remove such precursors, among which the combined phospholipase A2 (PLA2) and cyclodextrin mixed with soybean meal in a water bath and it is found that the removal rate of phospholipids is more effective (Zhu and Damodaran, 2018).

In previous studies, it was found that the principle of removing beany flavor was mainly divided into two steps: hydrolyzing PLA 2 to decompose PL, and then removing the hydrolyzed product by forming an inclusion complex through β-cyclodextrin (β-CD). PLA2 selectively cleaves the ester bond of the acyl chain at the sn-2 position of PL and generates Lyso-PL and FFA. The product is a non-polar material. β-CD is a cyclic non-reducing oligosaccharide. Its special construction makes its inner cavity hydrophobic. β-CD and other cyclodextrins can form water-soluble inclusion complexes with insoluble non-polar substances. The water-soluble inclusion compound can be dissolved into the supernatant by a polar solvent such as deionized water, so as to achieve the effect of separation from soybean flour.

Additional studies have shown that after the application of Alcalase, papain or a combination of enzymes, the acidic subunits of β-conglycinin and glycinin disappear completely, resulting in the removal of some of the precursors of the beany flavor, thereby reducing the beany flavor (Meinlschmidt et al., 2016).

### Odor masking and transformation

As shown in the low right of Figure 1, some strains can produce new aldehydes with aroma after fermentation while reducing the content of compounds with beany smell. In this way, the new aroma can mask the original beany smell to further enhance the total aroma profile of the product. For example, phenylacetaldehyde produced by fermentation of *L. rhamose* L08 can bring floral and honey-like aromas (Pei et al., 2022). And the main contributors to the aroma profile of *A. aegerita* fermented soy beverages remain soy-derived compounds. At the same time, ethyl esters and lactones were produced during fermentation, which resulted in increased fruity, floral, and creamy/dairy aromas present in cheese aromas and altered the overall aroma of the samples (Nedele et al., 2022a). Other scents include milky, nutty, and more.

## Application of microorganisms to remove beany flavor

In recent years, fermentation has become the primary method employed in many studies to reduce the beany flavor due to its various advantages. Several strains are discussed below that have shown potential to remove or cover the beany flavor in bean protein-based products (Table 1).

**Table 1:** Application of fermentation methods for removing beany flavor.

	Matrices	Strains	Fermentation conditions	Method	Function	Sensory perception	References
*Lactobacillus*	Mung bean	LAB (*L. plantarum*)	37 ± 1°C 48 h	Biotransformation	Transform aldehydes into esters.	The esters give the fermented products a pleasant fruity odor.	Yi et al. (2021)
	*Lupinus angustifolius* L.	Five lactic acid bacteria	28°C 48 h	Decomposition and masking	Reduce aldehydes, especially hexanal, which possesses “green” odor; Create new pleasant aromatic compounds.	Increase sourness and “vinegar” odor; Reduce the “beany” flavor as well as the unpleasant off flavor.	Laaksonen et al. (2021)
	Pea flour (*Pisum sativum* L.)	*L. rhamnosus* L08	37°C 2 days	Decomposition and masking	Increase the variety of acids and esters; Reduce the unpleasant flavor compounds such as nonanal, decanal, octanal, 1-hexanol and 2-ethyl-1-hexanol; Produce phenylethyl aldehyde that could bring pleasant aromas.	Reduce the unpleasant beany flavor; Produce floral and honey-like aromas.	Pei et al. (2022)
	Pea protein isolates	*L. plantarum*, *L. casei* and mixed strains of probiotics	37°C 5, 10, 15, 20, 25, and 30 h Anaerobic conditions	Decomposition	Remove around 42% aldehyde and 64% ketone content; Produce a small amount of alcohol.	Decrease the overall unpleasant aroma and flavor intensity.	Shi (2020)
	Lupin protein extracts (LPE)	*L. plantarum* L1047 and *Pediococcus pentosaceus* P113	−	Decomposition and masking	Decrease the concentration of n-hexanal and prevent its re-formation; Change the aroma profile which may mask off-flavors.	The more pleasant odor of the fermented protein extracts, compared to the unfermented protein extracts is explained by its different aroma profile.	Schindler et al. (2011)
	Soymilk	*Lactobacilli* and *Streptococci*	37°C 12 h	Decomposition	Reduce or even eliminate the concentrations of volatile components that have been associated with the beany flavor of soymilk, such as methanol, acetaldehyde, ethanol, and hexanal.	The heat treatment applied to the soymilk in the present study would certainly cause a severe cooked flavor. Thus, the resulting fermented product would not have been suitable for sensory analysis.	Blagden and Gilliland (2005)
	Pea protein isolates (PPI)	*L. plantarum*	37°C 30 h Anaerobic conditions	Decomposition	Eliminate aroma compounds that belong to the aldehyde, ketone, and alcohol groups.	Reduce the off-flavor (“hay” and “green” like aroma); Improve the aroma profile.	Shi et al. (2021)
	Pea (*Pisum sativum*) protein extract	*L. plantarum* L1047 or *P. pentosaceus* P113	37°C 48 h	Decomposition and masking	Decrease the n-hexanal content; Reduce or mask undesirable green notes.	Improve the aroma profile.	Schindler et al. (2012)
	Lupin protein isolate	*Lactobacilli*	30°C (*L. parabuchneri*, *L. brevis*) 37°C (*L. helveticus*, *L. delbrueckii*, *L. sakei sub* sp. *carnosus*, *L. reuteri*, *S. xylosus*) 42°C (*L. amylolyticus*) 36–48 h	Decomposition and masking	Decrease the n-hexanal content; Reduce or mask undesirable green notes.	Reduce the intensity of characteristic aroma impression (pea-like, green bell pepper-like) from 4.5 to 1.0.	Schlegel et al. (2019)
Yeast	Okara	Yeast (*Lindnera saturnus*)	30°C 48 h Solid-state fermentation	Biotransformation	Aldehydes are reduced into alcohols by yeast alcohol dehydrogenase or oxidized into acids by yeast aldehyde dehydrogenase, and finally form esters *via* enzymatic reactions.	Transform the aroma profile of okara from a green, grassy off-flavor into a markedly fruity and sweet aroma.	Vong and Liu (2018)
	Soybean residue (okara)	Four “dairy yeasts” (*Geotrichum candidum*, *Yarrowia lipolytica*, *Debaryomyces hansenii* and *Kluyveromyces lactis*) and six “wine yeasts” (*Saccharomyces cerevisiae*, *Lachancea thermotolerans, Metschnikowia pulcherrima*, *Pichia kluyveri, Torulaspora delbrueckii*, and *Williopsis saturnus*)	30°C 72 h Solid-state fermentation	Biotransformation	Oxidize the undesirable aldehydes into fatty acids, or reduce them into alcohols, and finally yield esters; Yeast proteinases and peptidases break down the protein in okara. Yeasts degrade the free amino acids to yield higher alcohols and esters.	The final fermented okara had a very strong fruity and estery character.	Vong and Liu (2017)
	Soy (tofu) whey	commercial non-*Saccharomyces* yeasts (*T. delbrueckii*; *L. thermotolerans*; *M. pulcherrima*; *P. kluyveri* and *W. saturnus*)	20°C 3 days	Decomposition & Masking	Metabolize endogenous carbonyls and alcohols to low or trace levels; Produce new alcohols, esters and acids to enrich aroma profiles that were unique to each non-*Saccharomyces* yeast.	Each yeast produced different levels of different volatile compounds that can contribute to the different aroma profiles of the fermented whey.	Chua et al. (2018)
Edible Basidiomycetes	Soybean products (soy drink and soy protein isolate)	*Lycoperdon pyriforme*	24°C 28 h In darkness	Biotransformation	Saturated aldehydes were metabolized by the fungus to their corresponding alcohols; Di-unsaturated aldehydes were synthesized to a non-volatile product.	Impart slightly bitter almond-like, fungal and nutty odor notes without recognition of the soy product off-flavors.	Nedele et al. (2022b)
	Okara	*Wolfiporia cocos CGMCC 5.55*, *W. cocos CGMCC 5.528*, *W. cocos CGMCC 5.78* and *Tremella fuciformis CGMCC 5.466*	25 ± 1°C 7 days	Decomposition & Masking	Decrease the content of off-flavor compounds like hexanal; New aromatic compounds were generated.	All fermented products had very little characteristics of beany flavor, and a fragrant, floral, and sweet aroma was present.	Wang Z. et al. (2022)
	Soy drink	*Agrocybe aegerita*	24°C 75 h In darkness	Decomposition and masking	Many typical soybean off-flavor contributors were reduced in their intensity such as green aldehydes; Synthesize ethyl esters and lactone, which change the overall aroma of the sample.	Produce a natural and vegan cheese aroma; Decrease the off-flavor in soybean-based products; Produce a sweet, floral and fruity flavor impression.	Nedele et al. (2022a)
	Soy drink	*L. pyriforme*	24°C 28 h In darkness	Decomposition & Masking	Increase aroma compounds such as 1-octen-3-one with a mushroom-like odor and benzyl alcohol with a sweetish flavor; Decrease most of the key aroma compounds with a green off-flavor.	The aroma of soy drink turned from green, beany, and oat-like to oat-like, mushroom-like, and almond-like.	Nedele et al. (2021)
Co-fermentation	Soybean (soymilk)	Three isolated new yeasts (*K. marxianus* SP-1, *Candida ethanolica* ATW-1, and *P. amenthionina* Y) and a commercial yeast (*K. marxianus* K) along with five strains of lactic acid bacteria (LAB)	36°C 5 h LAB and yeast ratio (5:2, v/v)	Biotransformation	Transform aldehydes into either acids, alcohols and esters.	Remove the beany flavor; Produce rich aromatic components; Improved the flavor and taste of drinks.	Korma et al. (2022)
	Okara (soybean residue)	Probiotic (*L. paracasei*) and yeast (*L. saturnus*)	30°C 48 h (viable cell count ratio of probiotic:yeast was about 100:1)	Biotransformation	Produce large amounts of esters to give a natural fruity aroma.	Give a natural fruity aroma.	Vong and Liu (2019)
	Pea protein-based product	Lactic acid bacteria and yeasts	pH 7.1 ± 0.1 to 4.55 30°C to 4°C	Decomposition & Masking	Degrade many off-flavor compounds; Trigger the generation of esters compounds with fruity and floral notes.	Reduce the concentration of pea off-notes; Generate new notes that could modify the perception of sensory defects; Improve the aroma quality of fermented beverages.	El Youssef et al. (2020)
	Okara	Compound probiotics (*L. plantarum*, *L. acidophiluss*, *Bifidobacterium lactis*, *L. casei* and *B. longum*, *S. cerevisiae* and *Hansenula* sp.) and mixed yeast (*S. cerevisiae* and *Hansenula* sp.)	28°C for 1 day 37°C for 2 days Anaerobic conditions Solid-state fermentation	Biotransformation	Convert aldehydes into alcohols and esters, among other compounds to improve the flavor of okara.	Give the okara a pleasant smell and taste. Sensory acceptability is greatly improved compared to unfermented okara.	Shi et al. (2020)

### Lactobacillus

Lactic fermentation might be a promising strategy to improve the aroma profile of plant-based food products as it results in a reduction or covering of undesirable flavors. Some *Lactobacillus* strains can reduce or eliminate aroma compounds that have been linked with the beany flavor which are members of the aldehyde, ketone, and alcohol groups. Several authors demonstrated how fermentation with lactic acid bacteria in pea, lupin protein extract, and soymilk, respectively, reduced the concentration of hexanal, which is primarily responsible for the greeny and beany off-flavor (Blagden and Gilliland, 2005; Schindler et al., 2011, 2012; Shi, 2020). Other researches have shown that lactic fermentation can produce higher aldehydes, alcohols, acids, and ester compounds through further biotransformation to cover the beany flavors (Schindler et al., 2011; Shi et al., 2021; Yi et al., 2021). Lipid degradation, which also contributes to the creation of the odors found in fermented foods, takes place concurrently with this biotransformation. For instance, *L. rhamnosus* L08 fermentation produced phenylethyl aldehyde that could bring floral and honey-like aromas, phenylethyl alcohol that exhibits a fresh bread-like, rose-like aroma, and several esters with floral and fruity fragrances, which had the effect of covering undesirable flavors (Pei et al., 2022).

### Yeast

Some yeasts are also used to modify the odor characteristics of plant-based products by biotransformation. The products of yeast fermentation often carry a pleasant flavor profile of alcohols and esters. Chua et al. fermented soy whey samples using five commercial non-*Saccharomyces* yeasts. Volatile compounds such as ethanol and 2-phenylethanol were found in the fermented products, giving them a rose-like aromatic character (Chua et al., 2018). Other studies used yeasts to ferment okara and obtained a very strong fruity and estery character since most of the undesirable aldehydes were reduced into alcohols and esters by the yeasts fermentation (Vong and Liu, 2017; Vong and Liu, 2018).

Unfortunately, although fermentation with lactic acid bacteria or yeasts reduces the beany flavor substances, the fermentation process of certain strains inevitably produces acids such as lactic acid and hexanoic acid, causing strong sour and wine flavors, which to some extent aggravate the undesirable flavor of the bean protein-based product (Laaksonen et al., 2021; Nedele et al., 2021; Wang Z. et al., 2022). Thus, the optimization of fermentation conditions is required to prevent the creation of unpleasant flavor compounds.

### Edible basidiomycetes

In the food processing industry, edible fungi are common fermentation strains. Among them, basidiomycetes have garnered increasing interest due to their abundance, diversity, accessibility, and nutritional benefits (Mahboubi et al., 2017; Sun et al., 2020; Bentil, 2021). While most conventional starter cultures mainly produce primary metabolites, basidiomycetes are noted for their sensory modification of various food products (Nedele et al., 2021; Rigling et al., 2021; Nedele et al., 2022a). They are able to modify the flavor because their highly sophisticated secretomes produce abundant natural flavor molecules (Bouws et al., 2008). The scientists used four types of edible fungi to reduce the beany flavor and gain new aromatic flavors of okara. These new flavors contain benzene, ethanol, and linalool, which are probably byproducts of enzymatic events occurring during the growth and metabolism of edible fungi. In addition to adding floral, sweet, and orange fragrances, the presence of these compounds can disguise the flavor characteristics of some unwanted components (Wang Z. et al., 2022). Apart from that, recent studies have confirmed that by using a unique fermentation procedure with *Lycoperdon pyriforme*, the beany flavor of soy beverages was diminished while the nutritional profile was maintained. During this fermentation process, aldehydes, the main off-flavor contributors, were decreased and some pleasant aroma components were created, imparting the finished product an almond- and nutty-like smell (Nedele et al., 2021, 2022b). Therefore, it can be expected that the edible basidiomycetes are likely to be a promising strain for flavor improvement in plant-based foods. Further research is required to ascertain which kind of strains can successfully grow in the various sources of legume dietary fiber systems as well as what fermentation conditions can facilitate the aroma profile improvement.

## Conclusion and outlook

In summary, oxidation of unsaturated fatty acids and hydrolysis of lipids are the primary causes of undesirable flavor in a plant protein-base food. At present, masking, biotransformation and enzymatic degradation are the main mechanisms and approaches for removing the beany flavor. Several species of microorganisms including lactobacillus, yeast, and edible basidiomycetes were demonstrated to reduce the level of beany flavor compounds by converting them into less impacting compounds or covering them with new pleasant compounds formed during fermentation. However, different fermentation approaches will produce different final flavor characteristics due to the different metabolic pathways and capacities of the strains. Thus, the strains utilized have a significant impact on how the fermentation affects the fragrance profile of the plant proteins. It should be noted that no technique is perfect, and each method has its own inherent advantages and drawbacks.

In order to further improve the flavor quality of plant-based meat analogs, future product development and application research can mainly focus on the following aspects: (a) Fermentation using some strains may produce products with high acidity, which may not be preferred by consumers. More research is needed to select suitable strains and optimize fermentation process to meet the preferences of consumers (Laaksonen et al., 2021). (b) Aside from legume proteins, other ingredients including microalgae, konjac, and edible mushrooms could be promising substitutes for meat analogs due to their superior production capabilities and high nutritional content. Scientists can process these ingredients using fermentation method to explore the next generation of meat analogs (Jiménez-Colmenero et al., 2012; Fu et al., 2021; Yuan et al., 2022). (c) The fermentation process produces amino acids, sugars, and a series of precursors for Maillard reactions. Therefore, if the fermentation process is properly designed and oriented, it will effectively promote the Maillard reaction, providing better flavor and taste to plant-based meat analogs and eliminating the beany flavor. (d) The metabolomic pathway of fermentation process should be further investigated to demonstrate how the beany flavor is decomposed with the aid of mass spectrometry, nuclear magnetic resonance or isotopic labeling experiments (Ran et al., 2022). And the influence of more volatile compounds exist at low amounts should be further studied to confirm their effect on the aroma change of the fermented product. Improvements of current fermentation techniques will benefit from the results of such research. (e) There is still a lack of comprehensive research on the application of fermentation to remove beany flavor from plant-based meat analog. Not only the technological aspects, but also the safety concerns should be taken into consideration. Possible safety problems such as microbiological stability, allergenic potential and heavy metal and toxic substance contamination during fermentation should be investigated to develop safe and efficient fermentation protocols for the production of plant-based meat analogs.

## Author contributions

XY and TZ contributed to conception and design of the study. AT, HZ, and JD wrote the first draft of the manuscript. YX, YL, JL, and JH helped review and revise the manuscript. All authors contributed to the article and approved the submitted version.

## Funding

This work was supported by the Science and Technology Development Funds, Macau SAR (0024/2022/A and 0004/2021/ITP), the Science and Technology Planning Project of Guangdong Province (2020B1212030008), Innovation Cultivation Project of Zhuhai College of Science and Technology (2019XJCQ006), and the Open Fund of Guangdong-Hong Kong-Macao Joint Laboratory for Contaminants Exposure and Health (GHMJLCEH-05).

## Conflict of interest

JL and JH were employed by Macau Uni-Win Biotechnology Co., Ltd.

The remaining authors declare that the research was conducted in the absence of any commercial or financial relationships that could be construed as a potential conflict of interest.

## Publisher’s note

All claims expressed in this article are solely those of the authors and do not necessarily represent those of their affiliated organizations, or those of the publisher, the editors and the reviewers. Any product that may be evaluated in this article, or claim that may be made by its manufacturer, is not guaranteed or endorsed by the publisher.
